# ACROMORFO study: gait analysis in a cohort of acromegalic patients

**DOI:** 10.1007/s40618-024-02340-3

**Published:** 2024-02-28

**Authors:** V. Cimolin, C. Premoli, G. Bernardelli, E. Amenta, M. Galli, L. Donno, D. Lucini, L. M. Fatti, B. Cangiano, L. Persani, G. Vitale

**Affiliations:** 1 Department of Electronics, Information and Bioengineering, Politecnico di Milano, Piazza Leonardo da Vinci 32, 20133 Milan, Italy; 2 IRCCS Istituto Auxologico Italiano, San Giuseppe Hospital, Strada Luigi Cadorna 90, 28824 Piancavallo, Italy; 3 Department of Medical Biotechnology and Translational Medicine, University of Milan, 20122 Milan, Italy; 4 DISCCO Department, University of Milan, 20122 Milan, Italy; 5 Exercise Medicine Unit, Istituto Auxologico Italiano, IRCCS, 20135 Milan, Italy; 6 Department of Endocrine and Metabolic Medicine, IRCCS, Istituto Auxologico Italiano, 20149 Milan, Italy; 7 Laboratory of Geriatric and Oncologic Neuroendocrinology Research, IRCCS, Istituto Auxologico Italiano, 20145 Milan, Italy

**Keywords:** Acromegaly, Gait analysis, Arthropathy, Quality of life, Risk of fall

## Abstract

**Purpose:**

In acromegaly, skeletal complications resulted to be associated with low quality of life (QoL) and high risk of falls. The aim of the present study was to perform a quantitative assessment of movement through gait analysis technique in patients with acromegaly.

**Study population:**

Thirty-three acromegalic patients [9 with active disease (AD), 14 with controlled disease (CD) and 10 with disease remission (RD)] and 20 healthy subjects were enrolled for the study.

**Measurements:**

Kinetic and kinematic data were collected with 3D-gait analysis. Kinematic data were processed to compute the Gait Profile Score (GPS), a parameter that summarizes the overall deviation of kinematic gait data relative to unaffected population.

**Results:**

The acromegalic group showed longer stance phase duration (*p* < 0.0001) compared to controls. The GPS and several gait variable scores resulted to be statistically higher in the acromegalic group compared to healthy controls. GPS values were significantly higher in AD compared to CD (*p* < 0.05) and RD groups (*p* = 0.001). The AD group presented significantly higher values in terms of hip rotation and ankle dorsiflexion compared to CD and RD groups and with regard to the foot progression compared to RD. Interestingly, patients with RD exhibited a more physiological gait pattern.

**Conclusion:**

Acromegalic patients showed quantitative alterations of gait pattern, suggesting instability and increased risk of falls. Arthropathy, along with its associated abnormal joint loading, proprioceptive impairment and hyperkyphosis could be contributing factors. Disease control and remission appear to improve postural balance. A better knowledge on walking performance in acromegaly would help to develop specific rehabilitation programmes to reduce falls’ risk and improve QoL.

## Introduction

Acromegaly is a rare disease characterized by growth hormone (GH) and insulin-like growth factor type I (IGF-I) hypersecretion, primarily due to somatotroph pituitary adenomas. Chronic exposure to high GH and IGF-I levels leads to the development of several complications associated with reduced life expectancy such as cardiovascular, neoplastic, respiratory or metabolic diseases [1–3]. Acromegalic arthropathy and osteopathy with high risks of vertebral fractures (VFs) heavily affect subjects’ quality of life (QoL) and cause functional disability [4–10].

VFs develop more frequently in the thoracic spine, and they are often anterior wedge fractures, contributing to kyphosis and back pain. Hyperkyphosis can increase spinal mechanical load, leading to relevant compressive forces on vertebral bodies, thereby increasing the risk of fractures [11, 12]. In addition to the mentioned complications, acromegaly exhibits other well-known characteristics, such as acral growth and deformity, and alterations in body composition due to the expansion of body water and weight gain, which can also have detrimental effects on the musculoskeletal system and walking performance [13].

In the literature, there are only a few studies that have evaluated posture and balance in acromegaly. Atmaca et al*.* studied static and dynamic balance and fear of falling in 48 patients with acromegaly. They observed an impairment of dynamic balance, postulating an alteration of proprioception [13]. Lopes et al. performed a postural and balance evaluation using the photogrammetry and stabilometry in a small cohort of acromegalic patients, both with controlled and active disease. Compared to healthy subjects, patients with acromegaly displayed impaired static balance and significant postural abnormalities. It was suggested that impaired balance mechanisms were the cause of them adopting different postures to compensate [14]. More recently, Homem et al*.* evaluated 17 acromegalic patients and 20 healthy subjects with balance scales, force platform and knee isokinetic dynamometry tests. They reported significant differences between the groups on several balance and gait scales, indicating worse performance and an increased risk of falls in acromegalic group [15]. However, until now, no studies have addressed the assessment of gait in patients with acromegaly.

In the last years, new biomedical engineering technologies have been developed for the quantitative assessment of human walking patterns. The widespread use of accelerometers and their integration into wireless embedded platforms allow for movement detection. Moreover, electromyography, using surface electrodes, can record the activity of selected muscles during movement [16, 17]. Nowadays, gait analysis has become an important tool in many clinical settings to select the most appropriate treatment and rehabilitation programme for neurological movement disorders, such as infantile cerebral palsy, amyotrophic lateral sclerosis, Parkinson’s disease and multiple sclerosis. It also facilitates the assessment of QoL, health status, physical function and the risk of falling [18, 19].

The aim of this study was to quantitatively analyse movement using 3D gait analysis in patients with acromegaly at different disease stages, comparing them with a control group of healthy subjects.

## Materials and methods

### Study population

In this case–control study, we included 33 subjects (13 males and 20 females, aged 29–86 years) with acromegaly on regular follow-up at ‘IRCCS Istituto Auxologico Italiano’ in Milan.

The diagnosis of acromegaly was based on high serum GH levels, not suppressible after 75-g oral glucose load (OGTT), when available, and high plasma IGF-I levels for sex and age, signs and symptoms of disease and radiological evidence of pituitary adenoma. At diagnosis, 20 patients had a pituitary microadenoma, whereas 13 had a macroadenoma. At enrolment, nine patients were in active disease (AD), whereas ten had previously undergone transsphenoidal surgery (TNS) with or without radiotherapy and were considered to be in remission of disease (RD) on the basis of GH levels suppressible after OGTT and normal IGF-I for age and sex. Finally, 14 patients had disease control (CD) showing normal IGF-I for age and sex with ongoing medical therapy (somatostatin analogues and/or pegvisomant). Only three patients had a deficit of at least one pituitary axis and were on adequate replacement treatment.

Exclusion criteria were the existence of severe cardiorespiratory, neurological or musculoskeletal disorders, previous orthopaedics surgery and previous lower-limb traumatic injuries.

A control group of 20 healthy individuals (10 females and 10 males, aged 31–72 years) was recruited from the staff of Politecnico di Milano. These subjects were not affected by any neurological or musculoskeletal disorders that could impact their gait abilities, and they were not taking any medications for chronic pain.

The study protocol was approved by the Ethics Committee of Istituto Auxologico Italiano (EC number: 2019_06_18_05), and all patients gave informed consents for the use of the anonymized clinical and biochemical data for research and publication purposes. The study was carried out in compliance with the World Medical Association Declaration of Helsinki and its later amendments.

### Hormone assays

Serum GH concentrations were measured using an automated chemiluminescence immunoassay by IDS-iSYS (Immunodiagnostic Systems, Boldon, UK). The reportable range of the assay is 0.050–100 ng/mL. Sensitivity of the assay was 0.015 ng/mL. Intra- and interassay coefficients of variation were 5.9 and 10.4%, respectively. The calibrators of this kit are traceable to the WHO International Standard for Somatropin from NIBSC, code 98/574.

Serum IGF-1 concentrations were measured using an automated chemiluminescence immunoassay by IDS-iSYS (Immunodiagnostic Systems, Boldon, UK). The reportable range of the assay is 10–1200 ng/mL. Sensitivity of the assay was 1.9 ng/mL. Intra- and interassay coefficients of variation were 5.4 and 7.2%, respectively. The calibrators of this kit are traceable to the WHO International Standard for IGF-I with the code 02/254.

### Kinematic and kinetic evaluation

All participants were evaluated with 3D gait analysis at the Movement Analysis Lab “Luigi Divieti” of the Department of Electronics, Information and Bioengineering, Politecnico di Milano, Milan (Italy), using an optoelectronic system composed of eight cameras (SMARTDX, BTS Bioengineering, Italy) set at 100 Hz and two force platforms (AMTI, USA).

To evaluate the kinematics of each body segment, passive markers were positioned on the participants’ body, as described by Davis et al. [20], and the underlying skeletal model was scaled on behalf of anthropometric data (height, weight, leg length, distance between the femoral condyles or diameter of the knee, distance between the malleoli or diameter of the ankle and distance between the anterior iliac spines and thickness of the pelvis). After placing the markers, the participants were asked to walk barefoot at self-selected speed along a walkway where the force platforms were embedded. Kinematic and kinetic data were collected for each subject from at least five trials to guarantee the reproducibility of the results. For each participant (both patients and controls), three trials consistent in terms of gait pattern (spatio-temporal, kinematic and kinetic) were considered for the analysis.

### Data analysis

The related positions of each joint and joint centre were estimated through the motion analysis and human anthropometric data. The limb rotation algorithm is based on the determination of Euler angles with a y–x–z axis rotation sequence. The joint rotation angles that are routinely obtained correspond to flexion/extension, adduction/abduction and internal/external rotation, respectively. Therefore, the joint rotation angles that are clinically determined are trunk and pelvic obliquity-tilt-rotation, hip adduction/abduction-flexion/extension-rotation, knee flexion/extension, ankle plantar/dorsiflexion and foot rotation. The trunk and pelvic angles are absolute angles, referenced to the initially fixed laboratory coordinate system; the hip, knee and ankle angles are all relative angles, e.g. the three hip angles describe the orientation of the thigh with respect to the pelvis and the foot rotation angle is an absolute angle, referenced to the laboratory, which indicates the position of the subject’s foot with respect to the direction of progression [20]. Kinematic data obtained from 3D gait analysis were normalized as a percentage of gait cycle, thus providing the trends of joint angle for pelvis, hip, knee and ankle, and they were processed to compute the Gait Profile Score (GPS), a parameter that summarizes the overall deviation of kinematic gait data relative to unaffected population as described by Baker et al. [21]. From a mathematical point of view, the GPS represents the root mean square (RMS) difference between the individual’s joint curve and the average curve calculated for a reference population of unaffected individuals. The overall GPS is based upon 15 clinically important kinematic variables (pelvic tilt, obliquity and rotation, hip flexion, abduction and internal rotation, knee flexion, dorsiflexion and foot progression for left and right sides) which are expressed as Gait Variable Scores (GVSs), each of which represents the RMS difference between a specific time-normalized gait variable and the mean data of a population of healthy individuals. The GPS is the RMS average of the GVS variables [Eq. 1]:

1 $${\text{GPS}}=\sqrt{\frac{1}{N} \sum_{i=1}^{N}{{\text{GVS}}}_{i}^{2}}$$

In this analysis, a GPS score for each side was used based on all nine GVSs for that side. As the GPS represents the difference between the patient’s data and the average from the reference dataset, the higher the GPS value is, the lower the physiological gait pattern. GPS values for unaffected individuals lie in the range of 5–6° [22–24]. The main spatio-temporal parameters (gait speed, step length, cadence, stance duration and step width) were calculated; as for step length, the normalized step length (normalized with respect to individual’s height) was reported. In this study, the kinetic data were not analysed, even if acquired.

### Statistical analysis

Statistical analysis was carried out using the Minitab (version 19.2020.1, State College, PA: Minitab, Inc.) software. All the parameters were computed bilaterally for each participant. All the parameters of interest resulted normally distributed (after the Shapiro–Wilk test), and a parametric statistic was used. We preliminarily checked all data separately acquired for left and right limbs to verify the presence of statistically significant differences between them. As they were not found, in the subsequent analysis for each participant, the limbs were considered independently.

First, *t* test for independent groups was performed to compare acromegalic group and control group. Then, one-way ANOVA was used to compare the three acromegalic subgroups and the control group. Furthermore, post hoc-analysis was performed to assess the contribution of each group in the variance of the spatio-temporal parameters and Gait Profile Score (GPS) and its Gait Variable Scores (GVSs). The ANCOVA test was performed to assess whether age, sex and body mass index (BMI) could act as potential confounding factors in the observed differences between acromegalic patients and controls. The Chi-square test was employed to compare the prevalence of disease complications amongst the three subgroups of acromegalic patients (AD, CD and RD). The level of significance was set at *p* < 0.05. Data were presented using percentages for categorical variables and mean ± standard deviation (SD) for continuous variables.

## Results

### Characteristics of patients with acromegaly and healthy subjects

We enrolled 33 acromegalic patients and 20 healthy individuals. No significant differences were observed between both groups in terms of age and gender. BMI was significantly higher in acromegalic patients than that of controls. The demographic and clinical parameters of acromegalic and control groups are reported in Table 1.

**Table 1:** Characteristics of patients with acromegaly and healthy subjects

Demographic variables	Acromegalic group (*n* = 33)	Control group (*n* = 20)	*p*-values
Age (years)	62.6 ± 11.79	54.9 ± 11.09	0.07
Males	13 (39.4%)	10 (50%)	0.45
Females	20 (60.6%)	10 (50%)	
BMI (kg/m^2^)	29.6 ± 6.1	23.92 ± 4.72	0.018
Disease data			
Disease remission	10 (30.3%)		
Controlled disease	14 (42.4%)		
Active disease	9 (27.3%)		
Microadenoma	20 (60.7%)		
Macroadenoma	13 (39.3%)		
Hypopituitarism	3 (9.1%)		
Diabetes	11 (36.3%)		
Arthropathy	8 (24.2%)		
Osteoporosis	11 (33.3%)		
Arterial hypertension	24 (72.7%)		
SAS	9 (27.3%)		
Malignancies	12 (36.4%)		

Data are reported as mean ± SD or number (%)

*BMI* body mass index, *SAS* sleep apnoea syndrome

Nine patients had AD. Four of them were naïve to surgical, pharmacological and radiotherapeutic treatments. The other five patients received previous treatments but resulted not controlled with the ongoing medical therapy. Fourteen patients had CD by ongoing medical therapy. Ten patients achieved RD after TNS; two of them received radiotherapy and medical treatment after TNS. The three subgroups of acromegalic patients (AD, CD and RD) exhibited no significant differences in terms of age and gender, BMI and the prevalence of disease complications, as reported in Table 2.

**Table 2:** Characteristics of patients with acromegaly according to disease status

	Active disease (*n* = 9)	Controlled disease (*n* = 14)	Disease remission (*n* = 10)	*p*-values
Age (years)	59.8 ± 12.81	62.34 ± 12.47	60.47 ± 13.08	0.12
Males	5 (55.5%)	6 (42.8%)	2 (20%)	0.26
Females	4 (45.5%)	8 (57.2%)	8 (80%)	0.26
BMI (kg/m^2^)	31.6 ± 6.3	29.1 ± 6.5	28.4 ± 5.1	0.25
Hypopituitarism	1 (11.1%)	0 (0%)	2 (20%)	0.63
Diabetes	2 (22.2%)	8 (57.1%)	1 (10%)	0.09
Arthropathy	3 (33.3%)	3 (21.5%)	2 (20%)	0.75
Osteoporosis	3 (33.3%)	4 (28.6%)	4 (40%)	0.84
Arterial hypertension	7 (77.7%)	10 (71.4%)	7 (70%)	0.92
SAS	3 (77.7%)	5 (35.7%)	1 (10%)	0.34
Malignancies	3 (77.7%)	8 (51.1%)	1 (10%)	0.06

Data are reported as mean ± SD or number (%)

*BMI* body mass index, *SAS* sleep apnoea syndrome

In the AD group, GH levels (3.78 ± 2.8 ng/mL) were significantly higher than those observed in the CD group (1.09 ± 0.86 ng/mL, *p* = 0.006) and the RD group (0.87 ± 0.56 ng/mL, *p* = 0.004). In the AD group, IGF-1 levels (2.02 ± 0.880), expressed as upper limit of normal (ULN), were significantly higher than those observed in the CD (0.66 ± 0.20, *p* = 0.006) and RD (0.66 ± 0.22, *p* = 0.001) groups.

### Kinematic and kinetic results

Considering spatio-temporal parameters, the acromegalic group showed similar values compared to controls except for the stance phase duration that resulted longer in patients with acromegaly (*p* < 0.001), as reported in Table 3.

**Table 3:** The spatio-temporal parameters of acromegalic group and the control group with *p*-values

Spatio-temporal parameters	Acromegalic group	Control group	*p*-values
% stance (% gait cycle)	62.87 ± 2.10	58.95 ± 1.85	** < 0.001**
Gait speed (m/s)	1.04 ± 0.19	1.08 ± 0.17	0.088
Cadence (step/min)	106.78 ± 12.51	103.00 ± 8.30	0.675
Step length (m)	0.58 ± 0.07	0.59 ± 0.05	0.444
Step width (m)	0.08 ± 0.03	0.08 ± 0.05	0.883

Data are reported as mean ± SD

Significant *p* values are expressed in bold

Regarding kinematic data, the GPS (*p* < 0.001) and several GVS variables were statistically higher in the acromegalic group than those of controls (Table 4). Through a detailed analysis of the GVS data, the acromegalic group presented higher values in terms of the pelvic tilt (*p* = 0.02), hip rotation (*p* < 0.001), ankle dorsiflexion (*p* = 0.009) and foot progression (*p* = 0.003) compared to controls.

**Table 4:** GPS and GVS values of acromegalic group and control group with *p*-values

GPS and GVSs [degrees]	Acromegalic group	Control group	*p*-values
GPS	9.58 ± 2.34	7.61 ± 0.97	** < 0.001**
Pelvic tilt	7.47 ± 3.01	5.12 ± 3.28	**0.020**
Pelvic obliquity	2.16 ± 0.99	2.60 ± 0.93	0.118
Pelvic rotation	4.10 ± 1.12	4.70 ± 1.43	0.131
Hip flexion	8.70 ± 3.65	6.86 ± 3.50	0.063
Hip abduction	4.67 ± 2.56	5.41 ± 2.76	0.380
Hip rotation	15.79 ± 6.01	7.35 ± 4.37	** < 0.001**
Knee flexion	7.23 ± 3.00	5.87 ± 2.10	0.267
Ankle dorsiflexion	7.34 ± 2.94	5.63 ± 2.14	**0.009**
Foot progression	13.76 ± 8.69	7.74 ± 3.98	**0.003**

Data are reported as mean ± SD

Significant *p* values are expressed in bold

*GPS* Gait Profile Score, *GVS* Gait Variable Score

To evaluate whether the covariates such as age, gender and BMI have effects on gait parameters, the ANCOVA test was performed. The results indicated that the covariates age and gender were not significantly associated with gait parameters. However, BMI was found to be significantly related only to stance duration [F (1, 83) = 45.58, *p* < 0.001]. No other statistically significant results were observed in this analysis.

To understand whether the activity of disease could have an impact on gait pattern, we performed a sub-group analysis according to the disease status.

No statistical differences were found in terms of spatio-temporal parameters amongst the three pathological groups, as reported in Table 5. All the sub-groups displayed statistically longer stance phase duration than that of controls.

**Table 5:** Spatio-temporal parameters of the acromegalic sub-groups in relation to the state of disease and control group with *p*-values

Spatio-temporal parameters	Active disease	Controlled disease	Remission of disease	Control group	*p*-values
% stance (%gait cycle)	62.51 ± 2.21**	62.90 ± 2.38**	63.14 ± 1.58**	58.95 ± 1.85	** < 0.001**
Gait speed (m/s)	1.08 ± 0.13	0.98 ± 0.22	1.10 ± 0.17	1.08 ± 0.17	0.35
Cadence (step/min)	104.97 ± 9.79	105.99 ± 14.92	109.68 ± 10.62	103.00 ± 8.30	0.52
Step length (m)	0.61 ± 0.05	0.56 ± 0.06	0.59 ± 0.08	0.59 ± 0.05	0.07
Step width (m)	0.10 ± 0.03	0.07 ± 0.02	0.08 ± 0.03	0.08 ± 0.05	0.12

Data are reported as mean ± SD. *p*-values are related to ANOVA analysis. *p* values by post-hoc tests are reported as follows: ** = *p* < 0.05 vs. control group

Significant *p* values are expressed in bold

Considering kinematic data (Table 6), GPS values resulted to be significantly higher in the AD group compared to that observed in the CD and RD (*p* < 0.05) groups. GPS was higher in both AD and CD groups compared to controls (*p* < 0.05). Going into further detail, the AD group presented significantly higher values in terms of hip rotation and ankle dorsiflexion compared to the CD group and RD group. Finally, in the AD group, foot progression was significantly higher compared to that observed in patients with RD. Considering both spatio-temporal parameters and kinematic data, no significant differences have been observed between CD and RD groups.

**Table 6:** GPS and its GVS variables of the acromegalic sub-groups in relation to the state of disease and control group with *p*-values

GPS and GVSs [degrees]	Active disease	Controlled disease	Remission of disease	Control group	*p*-values
GPS	11.09 ± 1.78°*^	9.08 ± 2.22^	8.61 ± 2.48	7.61 ± 0.97	** < 0.001**
Pelvic tilt	9.77 ± 2.92^	7.71 ± 4.23^	7.77 ± 3.53^	5.12 ± 3.28	**0.001**
Pelvic obliquity	2.26 ± 0.92	2.00 ± 0.91	2.22 ± 1.00	2.60 ± 0.93	0.17
Pelvic rotation	3.85 ± 0.75	3.54 ± 1.39	4.75 ± 1.49	4.70 ± 1.43	0.08
Hip flexion	9.15 ± 3.67	8.44 ± 3.73	9.26 ± 3.69	6.86 ± 3.50	0.18
Hip abduction	5.31 ± 2.10	4.14 ± 2.65	4.17 ± 2.20	5.41 ± 2.76	0.16
Hip rotation	18.58 ± 6.41°*^	14.05 ± 5.34^	15.47 ± 7.88^	7.35 ± 4.37	** < 0.001**
Knee flexion	8.12 ± 3.24	6.88 ± 2.82	6.54 ± 7.88	5.87 ± 2.10	0.23
Ankle dorsiflexion	9.39 ± 3.58°*^	6.64 ± 3.05	5.76 ± 2.23	5.63 ± 2.14	** < 0.001**
Foot progression	16.97 ± 8.59*^	15.01 ± 7.26^	7.25 ± 5.04	7.74 ± 3.98	** < 0.001**

Data are reported as mean ± SD. *p*-values are related to ANOVA analysis

*p* values by post-hoc tests are reported as follows: ° = *p* < 0.05 vs. controlled disease; * = *p* < 0.05 vs. remission of disease; ^ = *p* < 0.05 vs. control group

Significant *p* values are expressed in bold

*GPS* Gait Profile Score, *GVS* Gait Variable Score

It is noteworthy that patients with RD exhibited a more physiological gait pattern. Specifically, statistically significant differences were observed only in pelvic tilt and hip rotation when compared to the control group. On the contrary, patients with AD and CD demonstrated higher values in GPS, pelvic tilt, hip rotation and foot progression compared to controls. In addition, the AD group displayed significantly higher ankle dorsiflexion compared to healthy subjects (Table 6).

No significant correlations were observed between IGF-1 and GH values at the time of evaluation, spatio-temporal parameters and GPS and GVS variables.

## Discussion

In the last years, several studies have highlighted the negative impact of acromegaly on QoL. Arthropathy and joint pain have a great importance on affecting individual perception of physical and mental health [7, 9, 10]. Moreover, an increased prevalence of VFs and hyperkyphosis has been reported in acromegalic patients [11–13]. Both these features may have a detrimental effect on spine sagittal balance and gait pattern, resulting in increased risk of falls. In literature, there are only few studies that evaluated posture and balance impairment in acromegaly. These studies observed an altered dynamic balance in these patients compared to controls, postulating an alteration of proprioception [13–15]. However, gait analysis has never been performed in acromegalic patients.

In this pilot study, we performed for the first time a quantitative assessment of movement through a 3D gait analysis in acromegalic patients compared to healthy subjects. From our analysis emerged a gait impairment in acromegalic patients. Looking into detail at spatio-temporal parameters, we observed that acromegalic group showed a longer stance phase duration compared to the control group. The stance phase, which accounts for approximately 60–62% of the entire gait cycle, is the period when the foot is in contact with the ground, and the limb is bearing weight. It begins with heel strike and concludes with toe-off of the same foot. During this phase, the leg supports the body’s weight in both the sagittal and frontal planes while facilitating forward movement. [18]. It is well known that an extended stance phase is associated with postural instability during walking and an increased risk of falls. Indeed, a prolonged stance phase should be considered a stabilizing adaptation related to fear [19].

Considering kinematic data, we evaluated the GPS and GVSs in acromegalic patients and in the control group. GPS was developed to summarize kinematic data and to facilitate the understanding of the results of gait analysis. Together with GVSs, which comprise nine gait variables for each side of the body, GPS can quantify deviations from a normal gait pattern. This approach provides a straightforward, objective and immediate assessment of the degree of gait impairment and how it differs from that of a population without gait alterations. The acromegalic group showed statistically higher GPS values compared to the control group. Regarding GVSs, we observed higher values for pelvic tilt (which represents the orientation of the pelvis in respect to the thighbones and the rest of the body), hip intra and extra-rotation, ankle dorsiflexion and foot progression in patients with acromegaly compared to healthy controls. All these alterations suggest a global gait impairment and instability during walking in patients with acromegaly.

In addition, we conducted an analysis by dividing the acromegalic patients into three subgroups based on their disease status. We compared spatio-temporal, kinetic and kinematic parameters from the gait analysis between the AD, CD and RD groups and controls to confirm the impact of this disease on walking impairment and to assess whether this complication is reversible after therapy of acromegaly. The higher GPS values observed in the AD group, compared to the other two groups of acromegalic patients and controls, indicate gait impairment in this subgroup of patients, which is likely related to the chronic exposure to high GH and IGF-1 levels. When examining individual variables, we observed higher values for hip intra and extra-rotation, ankle dorsiflexion and foot progression in the AD group. This suggests that pharmacological and surgical interventions for acromegaly have a positive impact on these parameters, restoring postural balance in these patients, particularly after remission of disease.

Therefore, we found quantitative alterations of gait pattern in a cohort of acromegalic subjects, more evident in patients with active disease. These alterations reflect instability during walking, probably leading to a high risk of falls. As well as reported in the literature, high risk of falls is associated with high risk of fracture and reduced QoL. Our findings support previous data on instability and increased risk of falls in patients with acromegaly [13–15].

Different factors may take part in determining the dynamic balance alterations that we observed. Arthropathy is a well-known disease complication and may be one of the causes of this impairment. On the contrary, an altered gait, with its consequent abnormal load on weight-bearing joints, may enhance arthropathy, configuring a detrimental vicious circle. Moreover, enthesopathy leading to proprioceptive impairment, hyperkyphosis caused by VFs and acral growth and deformity represent additional factors involved in the gait disturbance.

Some limitations of this case–controlled study need to be acknowledged. First of all, the rarity of the disease had an impact on the small sample size. Acromegalic patients exhibited a higher BMI compared to the control group, and it emerged as a potential confounder for stance duration. However, BMI did not significantly impact other kinetic data, as well as GPS and GVS variables, confirming a global gait impairment and instability during walking in patients with acromegaly. In addition, it is important to note that this study did not assess the impact of diagnostic delay on gait analysis parameters.

This pilot study could open a new chapter of further interesting investigations by expanding the study population, matching groups for BMI, incorporating the evaluation of fall risk through specific tools, assessing the impact of diagnostic delay and including de novo patients in multicentre longitudinal studies.

In conclusion, we have reported for the first time an impaired walking performance in patients with acromegaly. Our analysis emphasizes that changes in kinematic and kinetic parameters are particularly pronounced in patients with active disease. Disease control and remission appear to improve postural balance in acromegalic patients. In addition, gait analysis can assist healthcare professionals in developing personalized rehabilitation programmes, monitoring patient progress and evaluating treatment effectiveness. This approach aims to enhance mobility, movement and balance while reducing the risk of falls in these patients.

## Data Availability

The data that support the findings of this study are available from the corresponding author upon reasonable request.
